# Integrated multi-omics identifies MCRS1 as a causal hub linking aging, metabolic syndrome, and breast cancer progression

**DOI:** 10.1097/JS9.0000000000004592

**Published:** 2026-01-20

**Authors:** Yiying Tao, Xibing Ding, Wei Xuan, Hao Zhu, Xiaohua Liu, Yuqing Chen, Jialin Wu, Jing Du, Jie Tian, Guojun Qian

**Affiliations:** a Key Laboratory of Anesthesiology (Shanghai Jiao Tong University, Ministry of Education), Department of Anesthesiology, Ren Ji Hospital, Shanghai Jiao Tong University School of Medicine, Shanghai, China; b Department of Ultrasound, Renji Hospital, Shanghai Jiao Tong University School of Medicine, Shanghai, China

**Keywords:** aging, breast cancer, MCRS1, Mendelian randomization, metabolic syndrome

## Abstract

**Purpose:**

Aging and metabolic syndrome (MetS) are intertwined risk factors for breast cancer (BC), but the core molecular nexus integrating these states is unknown. This study aimed to identify and validate a causal driver at this intersection.

**Methods:**

We integrated transcriptomic datasets from BC, MetS, and aging cohorts to identify common dysregulated genes. Machine learning algorithms prioritized a core diagnostic signature. We used Mendelian randomization to infer causality and characterized the lead candidate using single-cell RNA sequencing and comprehensive preclinical validation.

**Results:**

Our analysis identified a 25-gene core at the intersection of BC, MetS, and aging. Machine learning distilled this to five hub genes that formed a highly accurate diagnostic nomogram. Critically, Mendelian randomization established MCRS1 as the sole causal risk factor for BC among these candidates. Single-cell analysis revealed that *Mcrs1* is predominantly expressed in proliferating cancer cells, where it drives a transcriptional program of enhanced cell cycle, senescence, and metabolic reprogramming. Accordingly, genetic knockdown of *Mcrs1* profoundly suppressed BC cell proliferation and invasion *in vitro*, and *in vivo* experiments using an orthotopic BC model in C57BL/6 mice demonstrated significantly reduced tumor growth.

**Conclusion:**

This study identifies MCRS1 as a central molecular hub that causally links aging and MetS to BC pathogenesis. MCRS1 is a validated driver of tumor progression and a high-performance biomarker, representing a potential target for therapeutic development, particularly in BC patients with metabolic comorbidities.

## Introduction

Breast cancer (BC) is the most commonly diagnosed malignancy and a leading cause of cancer-related mortality in women worldwide, with an estimated 2.3 million new cases and 666 000 deaths reported globally in 2022^[1]^, with its profound heterogeneity presenting formidable challenges to clinical management^[2,3]^. Beyond established genetic predispositions, metabolic dysregulation – particularly the cluster of conditions known as metabolic syndrome (MetS) – has emerged as a critical risk factor for BC, being associated with a 17% increase in incidence, a three-fold higher risk of recurrence, and nearly a two-fold increase in BC-specific mortality^[4–8]^. While individual mechanisms have been elucidated, such as the mitogenic effects of the hyperinsulinemia/IGF-1 axis or the pro-tumorigenic microenvironment shaped by adipokines and inflammatory cytokines^[9–13]^, an integrated understanding of the underlying molecular network that governs this clinical association remains elusive.

Aging represents the fundamental biological process at the intersection of oncology and metabolism^[14,15]^. As the single strongest risk factor for cancer, aging provides a fertile ground for tumorigenesis through hallmarks such as genomic instability, epigenetic reprogramming, and the chronic, low-grade inflammation termed “inflammaging”^[16,17]^. Crucially, these same hallmarks of aging, especially cellular senescence and its associated secretory phenotype (SASP), can also disrupt systemic metabolic homeostasis, which is central to the pathophysiology of MetS^[18]^. This establishes a vicious cycle wherein the aging process exacerbates metabolic dysfunction, which in turn aggravates the age-related, pro-cancerous milieu. This interplay forms a critical conceptual framework – the “aging–metabolism–cancer triangle” – that is key to understanding the rising incidence of BC in modern societies.

However, despite extensive research into these pairwise relationships, a critical knowledge gap persists: it is unknown whether a core molecular nexus exists that integrates signals from all three pathological states at the cellular level. Previous bioinformatic studies have been hampered by a limited scope, focusing either on single factors like obesity or on pan-cancer analyses, thereby failing to resolve the specific regulatory genes that connect MetS and BC within the explicit context of aging. Addressing this gap is paramount for developing diagnostic tools and therapeutic strategies that can precisely target the large patient population with metabolic comorbidities.

Here, we hypothesized that a key gene network, driven by aging and co-dysregulated in both MetS and BC, contains tractable targets for diagnosis and therapy. To test this hypothesis, we designed and executed a multi-layered, integrative study in accordance with the TITAN guideline^[19]^. We began by identifying candidate genes at the intersection of BC, MetS, and an established aging signature through deep mining of transcriptomic datasets. We then employed machine learning algorithms to pinpoint the most diagnostically relevant hub genes and develop a clinical nomogram. Critically, we used Mendelian randomization (MR) to infer a causal relationship between our lead candidate and BC risk, followed by single-cell RNA sequencing (scRNA-seq) to resolve its cellular localization and functional program within the tumor microenvironment. Finally, we validated the pro-tumorigenic function of this core gene through rigorous *in vitro* and *in vivo* experiments. This study aims to systematically uncover the core molecular mediator linking aging, MetS, and BC, offering a new target and rationale for confronting this deadly triad.

**HIGHLIGHTS**

- Aging-related genes link metabolic syndrome to breast cancer risk.
- Aging-related gene MCRS1 is a causal driver of breast cancer.
- MCRS1 drives tumorigenesis via senescence and metabolic pathways.
- Targeting MCRS1 may treat age-related metabolic-driven cancers.

## Methods

### Study design

**Figure 1.**
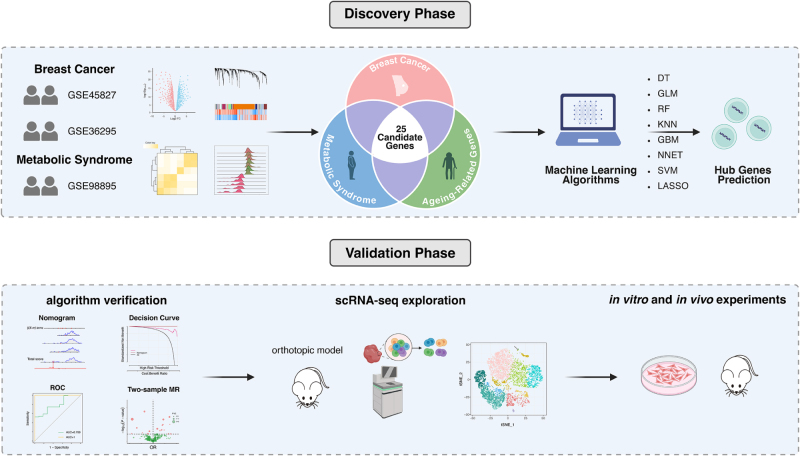
Schematic overview of the integrative discovery and validation pipeline. The study was conducted in two main phases. In the Discovery Phase, we first identified a common 25-gene signature by intersecting gene lists derived from transcriptomic analysis of breast cancer (BC; GSE45827 and GSE36295), metabolic syndrome (MetS; GSE98895), and a curated set of aging-related genes. This signature was then subjected to eight machine learning algorithms to prioritize a small number of high-confidence hub genes. In the Validation Phase, these hub genes were rigorously validated through three parallel approaches. First, their diagnostic utility and causal relationship with BC were assessed via algorithm verification (Nomogram, ROC, DCA) and two-sample Mendelian randomization (MR). Second, the cellular context and functional program of the lead candidate were explored using single-cell RNA sequencing (scRNA-seq) of an orthotopic mouse tumor model. Finally, the direct pro-tumorigenic functions were confirmed through comprehensive *in vitro* and *in vivo* experiments.

This study followed a systematic discovery-to-validation pipeline (Fig. 1). We first employed an integrative bioinformatic analysis of public transcriptomic data (BC and MetS) and curated gene lists (Aging) to identify a common gene signature, which was then refined to five hub genes using machine learning. Critically, we applied MR, using genetic variants as instruments for gene expression to minimize confounding and reverse causation, to nominate a causal lead candidate from these hubs. We then used scRNA-seq of a murine orthotopic tumor model to delineate the candidate’s cellular localization and functional programs within the tumor microenvironment. Finally, the pro-tumorigenic role of the candidate was validated experimentally through shRNA-mediated knockdown in a series of *in vitro* functional assays and an *in vivo* orthotopic xenograft model.

### Data acquisition and preprocessing

Publicly available transcriptomic datasets were retrieved from the Gene Expression Omnibus (GEO) database (http://www.ncbi.nlm.nih.gov/geo). These included two BC datasets (GSE45827^[20]^, Affymetrix Human Genome U133 Plus 2.0 Array, comprising 130 BC as well as 11 normal tissue samples and 14 cell lines; GSE36295^[21]^, Affymetrix Human Gene 1.0 ST Array, comprising 45 BC and 8 healthy tissue samples) and one MetS dataset (GSE98895^[22]^, Illumina HumanHT-12 V3.0, comprising 20 MetS and 20 healthy control samples). Raw data were processed using the limma package in *R*^[23]^. Background correction, quantile normalization, and probe summarization were performed. Probes were annotated to gene symbols, and for genes with multiple probes, the one with the highest average expression was retained. The aging signature gene set was curated from three comprehensive sources: the Human Ageing Genomic Resources “human aging genes” list^[24]^, the “human age-related genes” list from the Ageing Atlas database^[25]^, and a validated gene signature of cellular senescence from the literature^[26,27]^. This composite list represents genes robustly associated with cellular hallmarks of the aging process.

### Differential expression and gene set enrichment analysis

Differential expression analysis between disease and control groups for GSE45827 (tissue samples only) and GSE98895 was conducted using the limma package. Genes with an adjusted *P*-value of <0.05 and an absolute log2(Fold Change) of >1 were considered differentially expressed genes (DEGs). Gene set enrichment analysis (GSEA) was performed using the clusterProfiler package in *R* on the entire ranked list of genes^[28]^. The gene ontology (GO) and Kyoto Encyclopedia of Genes and Genomes (KEGG) gene sets from the Molecular Signatures Database (MSigDB) were used as references^[29–32]^. A Benjamini–Hochberg adjusted *P*-value of <0.05 was considered statistically significant^[33]^.

### Weighted Gene Co-expression Network Analysis

Weighted Gene Co-expression Network Analysis (WGCNA) was performed on the GSE36295 dataset using the WGCNA *R* package to identify modules of co-expressed genes associated with the BC phenotype^[34]^. A soft-thresholding power was selected to ensure a scale-free network topology. The adjacency matrix was transformed into a topological overlap matrix, and hierarchical clustering was used to identify gene modules. The module eigengene (the first principal component of each module) was correlated with clinical traits (BC vs. Control) to identify biologically significant modules.

### Identification of common genes and pathway enrichment

A four-way intersection analysis was performed to identify common genes among: (1) DEGs from GSE45827 (BC), (2) genes from the most significant WGCNA modules in GSE36295 (BC), (3) DEGs from GSE98895 (MetS), and (4) the curated aging signature. The resulting common gene list was subjected to pathway and process enrichment analysis using Metascape against the WikiPathways database^[35,36]^.

### Machine learning and hub gene selection

To identify key diagnostic markers, a suite of supervised machine learning models, including decision tree (DT)^[37]^, generalized linear model (GLM)^[38]^, random forest (RF)^[39]^, *K*-nearest neighbor (KNN)^[40]^, gradient boosting machine (GBM)^[41]^, neural network (NNET)^[42]^, support vector machine (SVM)^[43]^, and least absolute shrinkage and selection operator (LASSO)^[44]^, were trained on the GSE36295 dataset using the expression of the 25 common genes. The Caret package in *R* was used for model training and evaluation^[45]^. We employed a 10-fold cross-validation scheme with three repeats to tune model hyperparameters and obtain robust performance estimates. For models requiring tuning (e.g., SVM, NNET, and RF), we utilized a grid search approach to select the optimal parameters that minimized the root mean square error (RMSE). All other models were run with their default Caret package settings. Model performance was assessed based on the RMSE and the distribution of residuals. Feature importance was extracted from the best-performing model (GLM) to identify the top five hub genes.

### Construction and validation of the diagnostic nomogram

To build a more robust diagnostic model and mitigate the risk of overfitting, we first created an integrated training cohort. Transcriptomic data from GSE36295 and GSE45827 were combined, and batch effects were removed using the ComBat function from the sva *R* package^[46]^. A logistic regression model incorporating the five hub genes (MCRS1, MIF, PARP1, ADIPOR1, and ACD) was developed using the integrated dataset, and a corresponding nomogram was constructed.

For external validation, we utilized the independent GSE22820 dataset, which includes 176 BC and 10 normal breast tissue samples profiled on the Affymetrix Human Genome U133 Plus 2.0 Array^[47]^. The expression data were processed similarly to the training datasets. We then applied the trained nomogram model to the GSE22820 cohort to predict the probability of breast cancer for each sample and calculated the area under the curve (AUC) to assess its performance on this unseen data.

### MR analysis

A two-sample MR analysis was performed to infer the causal relationship between the five hub genes and BC risk. We first attempted to identify instrumental variables (IVs) from the Genotype-Tissue Expression (GTEx) v8 database for breast mammary tissue^[48]^. However, after stringent quality control, no genetic variants met the criteria (*P* < 5 × 10^−8^, *F*-statistic > 10) to serve as valid instruments for our hub genes in this tissue-specific dataset.

Consequently, we utilized the much larger eQTLGen consortium (*n* > 30 000, primarily whole blood) to ensure sufficient statistical power for instrument discovery^[49]^. For MCRS1, we identified a single independent, genome-wide significant SNP (rs80312011) that was strongly associated with its expression and served as a valid instrument (*F*-statistic = 36.47). Summary statistics for BC risk were obtained from the UK Biobank (ukb-a-55 and ukb-b-16 890, https://www.ukbiobank.ac.uk/)^[50]^. Given that only one valid IV was available for MCRS1, the causal effect was estimated using the Wald ratio method^[51]^, which is the ratio of the SNP-outcome effect to the SNP-exposure effect. As this method uses a single IV, sensitivity analyses for assessing horizontal pleiotropy (e.g., MR-Egger and Weighted Median) were not applicable.

### scRNA-seq analysis

Raw scRNA-seq data from the orthotopic E0771 tumor model were processed using the Seurat package in *R*^[52]^. Cells were filtered based on standard quality control metrics (nFeature_RNA, nCount_RNA, and percent.mt)^[53]^. Data were normalized, and principal component analysis was performed. Cell clusters were identified using the Louvain algorithm and annotated based on the expression of canonical marker genes^[54]^. The cancer cell cluster was subsetted, and differential expression analysis was performed between *Mcrs1*-positive and *Mcrs1*-negative subgroups. GSEA was then conducted on the resulting ranked gene list.

### Cell culture and gene knockdown

The murine BC cell line E0771 was obtained from the FuHeng Cell Center (Shanghai, China), and cultured in high glucose Dulbecco’s modified Eagle’s medium (DMEM, Gibco, Waltham, USA) supplemented with 10% fetal bovine serum (FBS, Gibco) and 50 U/mL penicillin and streptomycin (60162ES76, Yeasen Biotech, Shanghai, China). Cells were maintained at 37°C in an incubator under a humidified atmosphere of 5% CO_2_. Lentiviral particles containing shRNA targeting *Mcrs1* or a non-targeting control were used for transduction. Stable cell lines were selected and maintained in media containing puromycin (2 μg/mL). Knockdown efficiency was confirmed by qPCR and Western blot.

### In vitro *functional assays*

#### Proliferation

Cell viability was assessed using the Cell Counting Kit-8 (CCK-8) assay (40203ES60, Yeasen Biotech, Shanghai, China). For colony formation, cells were seeded in six-well plates and cultured for 10 days before staining with crystal violet.

#### DNA synthesis

The EdU Yefluor 594 In Vitro Kit (40276ES60, Yeasen Biotech, Shanghai, China) was used according to the manufacturer’s instructions. The proportion of EdU-positive cells was quantified by fluorescence microscopy.

#### Migration and invasion

Cell migration and invasion were assessed using Transwell chambers (8 μm pore size, Corning) with or without diluted Matrigel (1:8, Corning) coating. Cells that migrated/invaded to the lower surface were stained and counted.

### Western blot and immunofluorescence

For Western blotting, total protein was extracted, separated by SDS–PAGE, and transferred to PVDF membranes. Membranes were incubated with primary antibodies against MCRS1 (11362-1-AP), E-cadherin (3195S), N-cadherin (13116S), Vimentin (5741S), and GAPDH (60004-1-Ig), followed by HRP-conjugated secondary antibodies. For immunofluorescence, cells were fixed, permeabilized, and incubated with primary antibodies, followed by fluorescently labeled secondary antibodies. Nuclei were counterstained with DAPI.

### Animal studies

This work has been reported in accordance with the ARRIVE guidelines (Animals in Research: Reporting *In Vivo* Experiments^[55]^. All animal procedures were approved by the Institutional Animal Care and Use Committee (IACUC).

#### Animals

Female C57BL/6 mice (6–8 weeks old) were obtained from JieSiJie Laboratory Animal Co., Ltd, Shanghai, China. All animals were specific pathogen-free and housed in individually ventilated cages with corncob bedding, four animals per cage. Mice were maintained under controlled environmental conditions (12 h light/dark cycle, temperature 22 ± 2°C, relative humidity 50–60%) with free access to standard chow and water. Environmental enrichment (nesting materials and shelters) was provided. All animals were drug- and test-naïve prior to the study, weighting 18–22 g at the start of the experiment.

#### Study design and group allocation

Mice were randomly assigned to two groups (*n* = 8 per group): (1) vector group and (2) shMCRS1 group. Randomization was performed using a random number generator. Investigators responsible for tumor measurements were blinded to group allocation. The experimental unit was a single animal.

#### Procedures and timeline

On day 0, mice were anaesthetized with isoflurane (2% in oxygen, delivered via nose cone) and orthotopically injected into the fourth inguinal mammary fat pad with 1 × 10^6^ vector-control or shMCRS1 E0771 cells. No additional analgesia was required due to the minimally invasive procedure and rapid recovery from anesthesia. Tumor growth was monitored every 2 days using a caliper, and tumor volume was calculated using the formula: Volume = 0.5 × length × width^2^ from day 6. On day 15, mice were euthanized by CO_2_ inhalation followed by cervical dislocation, and tumors were excised, weighed, and photographed. The primary outcome was tumor growth rate, measured as tumor volume over time. Secondary outcomes included tumor weight and tumor size at endpoint.

#### Sample size and replication

The number of animals (*n* = 8 per group) was determined based on previous studies^[56,57]^ using similar orthotopic BC models, which showed this sample size provides sufficient statistical power to detect meaningful differences in tumor growth between groups. Each experiment was performed once with all animals included in the analysis.

#### Adverse events and protocol modifications

No unexpected adverse events occurred during the study. To minimize stress and potential complications, anesthesia was carefully monitored, and mice were returned to their home cages immediately after recovery from isoflurane. No further protocol modifications were required.

### Statistical analysis

Statistical analyses were performed using R software and GraphPad Prism. Tumor growth curves were compared using two-way ANOVA with repeated measures. For comparisons between two groups, a two-tailed Student’s *t*-test was used. Data are presented as mean ± standard deviation (SD). A *P*-value of <0.05 was considered statistically significant.

## Results

### The transcriptomic landscape of BC is characterized by dysregulation of aging and metabolic pathways

**Figure 2.**
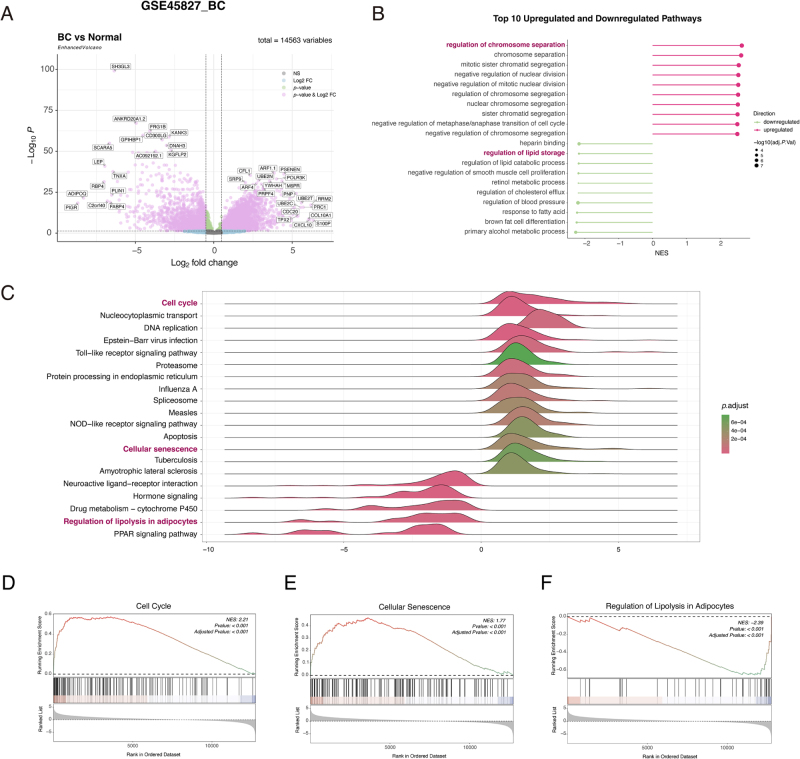
The breast cancer transcriptome is characterized by a dual signature of activated senescence and suppressed metabolic pathways. All panels are based on the analysis of the GSE45827 breast cancer (BC) dataset. (A) Volcano plot of differentially expressed genes (DEGs) in BC versus normal tissues. Genes with an adjusted *P* of <0.05 and |log2(Fold Change)| > 1 are highlighted in pink. (B) Gene set enrichment analysis (GSEA) of Gene Ontology (GO) terms. The plot displays the top 10 upregulated (pink) and downregulated (green) biological processes in BC. The *x*-axis represents the NES, and the size of the points corresponds to the −log_10_(adjusted *P*-value). (C) Ridge plot showing GSEA results for selected Kyoto Encyclopedia of Genes and Genomes (KEGG) pathways. The color scale indicates the adjusted *P*-value. (D–F) GSEA enrichment plots for the (D) “Cell Cycle” (NES = 2.21, adjusted *P* < 0.001), (E) “Senescence” (NES = 1.77, adjusted *P* < 0.001), and (F) “Regulation of Lipolysis in Adipocytes” (NES = −2.39, adjusted *P* < 0.001) pathways.

To define the molecular signature of BC, we first performed differential expression analysis on the GSE45827 transcriptomic dataset, which identified numerous DEGs (Fig. 2A). To understand the functional alterations underlying this global expression profile, we next performed GSEA.

Analysis using the GO database revealed a distinct functional polarization. Gene sets related to cell division and chromosome dynamics, such as “regulation of chromosome separation,” were significantly enriched among upregulated genes in BC tissue (Fig. 2B). Conversely, gene sets associated with metabolic functions, including “regulation of lipid storage,” were significantly enriched among downregulated genes (Fig. 2B).

We further specified these findings by performing GSEA with the KEGG pathway database. This analysis highlighted “Cell Cycle” and “Cellular Senescence” as two of the most significantly activated pathways in BC (Fig. 2C). Detailed examination confirmed a strong positive enrichment for the “Cell Cycle” (Normalized Enrichment Score [NES] = 2.21, adjusted *P* < 0.001; Fig. 2D) and “Cellular Senescence” (NES = 1.77, adjusted *P* < 0.001; Fig. 2E) gene sets. In contrast, the “Regulation of Lipolysis in Adipocytes” pathway, a process central to MetS, was significantly suppressed (NES = −2.39, adjusted *P* < 0.001; Fig. 2F).

Collectively, this transcriptomic analysis identified a dual signature in BC, defined by the concurrent activation of cell cycle and senescence pathways and the suppression of core metabolic pathways. This finding provided the basis for investigating the molecular intersection of BC, aging, and MetS.

### WGCNA identifies distinct gene modules associated with senescence and metabolism in BC

**Figure 3.**
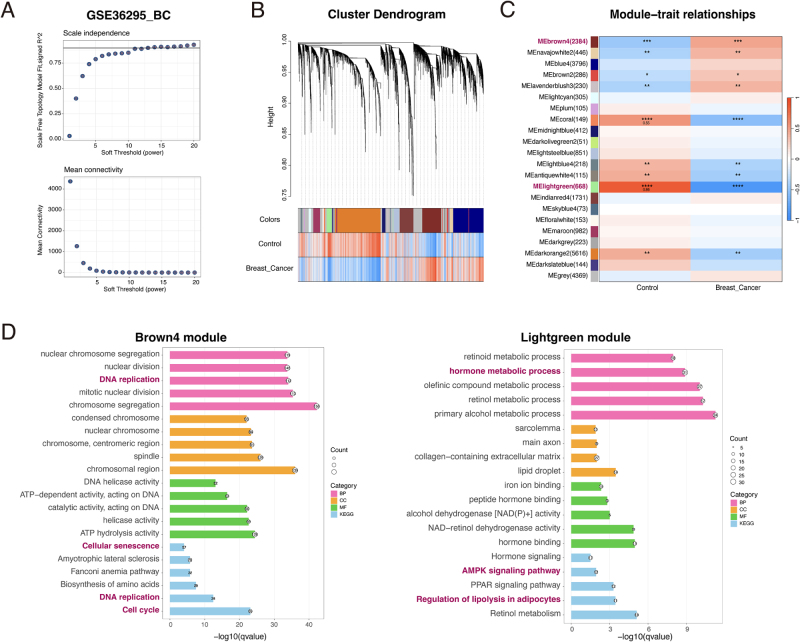
WGCNA identifies functionally distinct senescence and metabolism modules associated with breast cancer. All panels are based on Weighted Gene Co-expression Network Analysis (WGCNA) of the GSE36295 breast cancer dataset. (A) Determination of the soft-thresholding power (β). A power of 11 was selected to achieve a scale-free topology (*R*^2^ > 0.85) for network construction. (B) Hierarchical clustering dendrogram of genes. Identified co-expression modules are represented by the colors in the bar below the dendrogram. (C) Heatmap of module–trait correlations. The brown4 module shows the strongest positive correlation with the BC phenotype, while the light green module shows the strongest negative correlation. Red indicates positive correlation; blue indicates negative correlation. Significance is denoted by asterisks (*P < 0.05, **P < 0.01, ***P < 0.001, ****P < 0.0001). (D) Functional enrichment analysis (GO and KEGG) of genes within the positively correlated brown4 module (left), which is enriched for senescence and cell cycle pathways, and the negatively correlated light green module (right), which is enriched for metabolic pathways. BP, biological process; CC, cellular component; MF, molecular function.

To complement the DEG-based analysis and identify networks of co-expressed genes associated with the BC phenotype, we applied WGCNA to an independent dataset, GSE36295. A soft-thresholding power of 11 was selected to ensure a scale-free network topology (scale-free *R*^2^ > 0.85) while maintaining adequate mean connectivity (Fig. 3A). This analysis partitioned the transcriptome into 22 distinct co-expression modules (Fig. 3B).

We next correlated these modules with the clinical traits (BC vs. Control). This module–trait relationship analysis revealed two modules of primary interest. The brown4 module exhibited the strongest positive correlation with the BC phenotype, while the lightgreen module displayed the most significant negative correlation with BC (and conversely, a positive correlation with the control state) (Fig. 3C). These findings identified two robust gene networks whose collective expression is profoundly altered in BC tissue.

To determine the biological functions of these key modules, we performed functional enrichment analysis on the constituent genes. The genes within the positively correlated brown4 module were significantly enriched in biological processes and pathways related to cell proliferation and aging, including “DNA replication,” “Cell cycle,” and “Cellular senescence” (Fig. 3D, left panel). In contrast, the genes within the negatively correlated lightgreen module were predominantly enriched in metabolic pathways, such as “hormone metabolic process,” “AMPK signaling pathway,” and “Regulation of lipolysis in adipocytes” (Fig. 3D, right panel).

Thus, through an independent analytical approach on a separate patient cohort, we confirmed that the transcriptomic signature of BC is defined by the coordinated upregulation of an aging- and proliferation-associated gene network and the downregulation of a metabolism-centric network. This provided two distinct, functionally coherent sets of genes to further investigate for their convergent roles in BC pathogenesis.

### Identification of a core 25-gene signature at the intersection of BC, aging, and MetS

**Figure 4.**
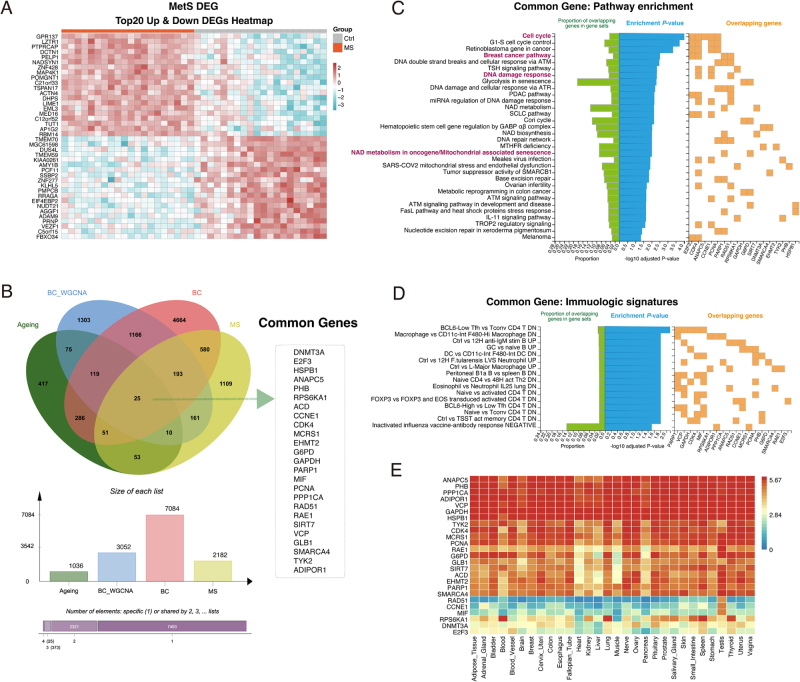
A 25-gene signature is identified at the intersection of breast cancer, aging, and metabolic syndrome. (A) Heatmap of the top 20 upregulated and downregulated DEGs between metabolic syndrome (MetS) patients and healthy controls from the GSE98895 dataset. (B) Venn diagram showing the four-way intersection of gene sets derived from BC DEGs (GSE45827), BC WGCNA modules (GSE36295), MetS DEGs (GSE98895), and a curated aging signature. This analysis yielded a core signature of 25 common genes. (C) Pathway enrichment analysis (WikiPathways) of the 25 common genes. The genes are significantly enriched in pathways related to the cell cycle, DNA damage response, and senescence. The right panel indicates which genes are present in each enriched pathway. (D) Immunologic signature enrichment analysis of the 25 common genes, indicating a role in inflammatory processes. (E) Heatmap showing the expression of the 25 common genes across various human tissues from the GTEx database. High expression is observed in breast and adipose tissue, among others. Color scale indicates log_2_(TPM + 1).

Having established that BC is linked to aging-related pathways and MetS, we next sought to identify the specific molecular nexus connecting all three conditions. First, we identified the DEGs between patients with MetS and healthy controls using the GSE98895 dataset (Fig. 4A).

We then performed a four-way intersection analysis to isolate a core set of common genes from (1) the DEGs identified in the primary BC dataset (GSE45827), (2) the clinically significant genes from the BC WGCNA modules (GSE36295), (3) the curated aging signature, and (4) the newly identified MetS DEGs. This stringent filtering process yielded a core signature of 25 common genes that are robustly associated with all three biological states (Fig. 4B).

To elucidate the collective function of this 25-gene signature, we performed pathway enrichment analysis. This analysis confirmed that the common genes were significantly overrepresented in pathways central to our hypothesis. Top enriched pathways included “Cell cycle” and “Breast cancer pathway,” alongside critical processes linking aging and cancer, such as “DNA damage response” and “NAD metabolism in oncogene/mitochondrial associated senescence” (Fig. 4C). Furthermore, these genes were also enriched in multiple immunologic signatures, pointing to a role in the chronic inflammatory state that is a shared feature of aging, MetS, and cancer (Fig. 4D).

The identification of this functionally coherent 25-gene signature, which is broadly expressed across various tissues including breast and adipose tissue (Fig. 4E), provided a high-confidence set of candidates for subsequent machine learning analysis to pinpoint the most critical diagnostic hub genes.

### Machine learning identifies five core hub genes from the common signature

To distill the most influential diagnostic markers from the 25-gene common signature, we employed a suite of supervised machine learning algorithms. We trained and evaluated multiple models, including DT, GBM, GLM, NNET, RT, SVM, KNN, and LASSO, on their ability to classify disease status based on the expression of these genes.

**Figure 5.**
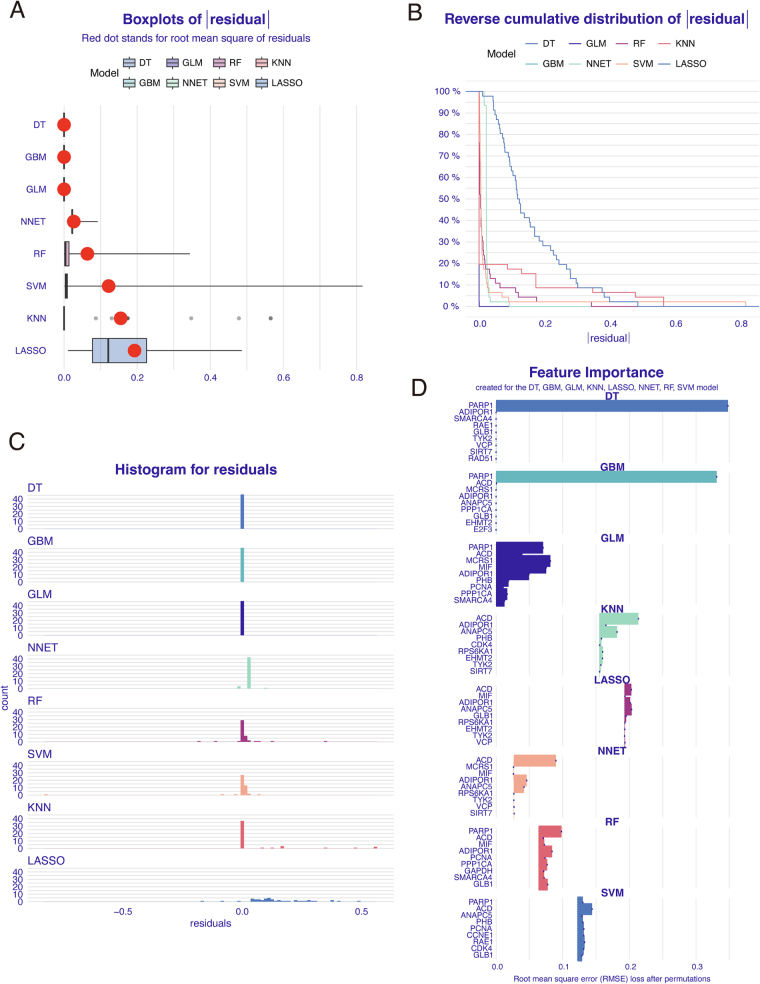
Machine learning identifies a five-gene hub signature with superior diagnostic performance. Multiple machine learning models were trained on the expression of the 25 common genes from the GSE45827 dataset to classify BC versus normal samples. (A) Boxplots of the absolute prediction residuals for each model. The red dot indicates the root mean square error (RMSE). (B) Reverse cumulative distribution of absolute residuals. (C) Histograms of the prediction residuals for each model. (D) Feature importance rankings for each model based on the RMSE loss after permutations.

The performance of each model was rigorously assessed by analyzing the distribution of prediction residuals. The GLM model demonstrated superior predictive accuracy, characterized by one of the lowest RMSEs and a highly compact residual distribution centered tightly around zero (Fig. 5A–C). Furthermore, the reverse cumulative distribution plot confirmed the robustness of the GLM, showing that its prediction errors were minimal for nearly all samples (Fig. 5A–C).

Having established the superior performance of the GLM, we interrogated its feature importance rankings to identify the genes with the highest predictive weight. This analysis pinpointed a concise set of five genes that were most critical for the model’s classification accuracy: MCRS1, MIF, PARP1, ADIPOR1, and ACD (Fig. 5D, GLM panel). These five hub genes were therefore selected for the development of a diagnostic nomogram and for subsequent investigation into their causal relationship with BC.

### A five-gene nomogram exhibits exceptional performance for BC diagnosis

**Figure 6.**
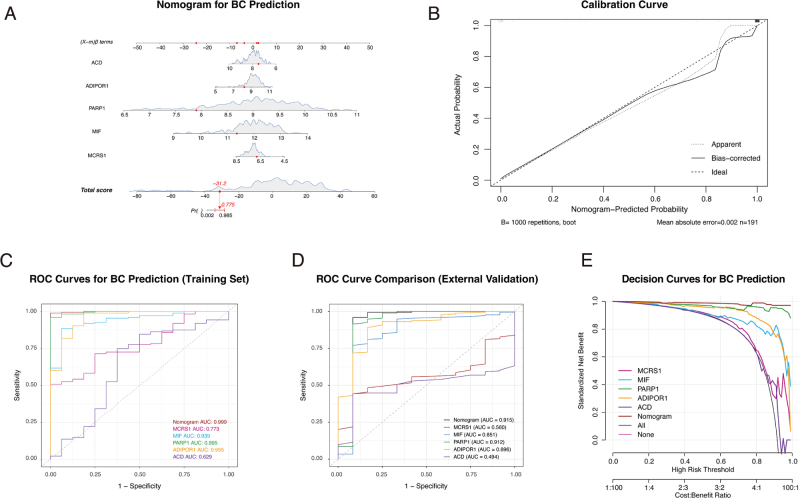
Development and external validation of a five-gene nomogram for breast cancer diagnosis. (A) A diagnostic nomogram constructed to predict the probability of breast cancer (BC) based on the expression of five hub genes (ACD, ADIPOR1, PARP1, MIF, and MCRS1). (B) Calibration curve of the nomogram evaluated in the training cohort. The bias-corrected line (solid) closely follows the ideal diagonal line (dashed), indicating excellent agreement between nomogram-predicted probabilities and actual outcomes. (C) Receiver operating characteristic (ROC) curve analysis of the nomogram and individual genes in the training set. The nomogram demonstrated near-perfect discrimination with an area under the curve (AUC) of 0.999. (D) ROC curve analysis in the independent external validation set. The nomogram maintained robust and excellent performance with an AUC of 0.915, confirming its generalizability and mitigating concerns of overfitting. (E) Decision curve analysis (DCA) illustrating the clinical utility of the nomogram. In the training cohort, the nomogram (brown line) provided a superior net benefit across a wide range of clinically relevant risk thresholds compared to any single gene or the default strategies of treating all or no patients.

To translate our findings into a clinically applicable tool, we constructed a diagnostic nomogram based on the expression levels of the five hub genes (MCRS1, MIF, PARP1, ADIPOR1, and ACD) using an integrated training cohort (GSE36295 and GSE45827) (Fig. 6A). The nomogram provides a simple, graphical method to calculate a total score, which corresponds to the predicted probability of a patient having BC.

The performance of the nomogram was rigorously evaluated. Within the training cohort, the model demonstrated excellent calibration, with the bias-corrected calibration curve showing strong concordance between predicted probabilities and actual outcomes (Fig. 6B). The ROC curve analysis in the training set also showed high discriminative power, achieving an AUC of 0.99 (Fig. 6C).

Critically, we then tested the nomogram’s generalizability on a completely independent external dataset, GSE22820. The model maintained strong diagnostic performance, achieving an AUC of 0.915 in this validation cohort (Fig. 6D). Finally, decision curve analysis (DCA) demonstrated that the nomogram provided a superior net benefit across a wide range of clinically relevant risk thresholds compared to alternative strategies in the training cohort (Fig. 6E). These results establish our five-gene signature as a robust and externally validated biomarker panel with high potential for BC diagnosis.

### MR identifies MCRS1 as a causal risk factor primarily expressed in proliferating cancer cells

**Figure 7.**
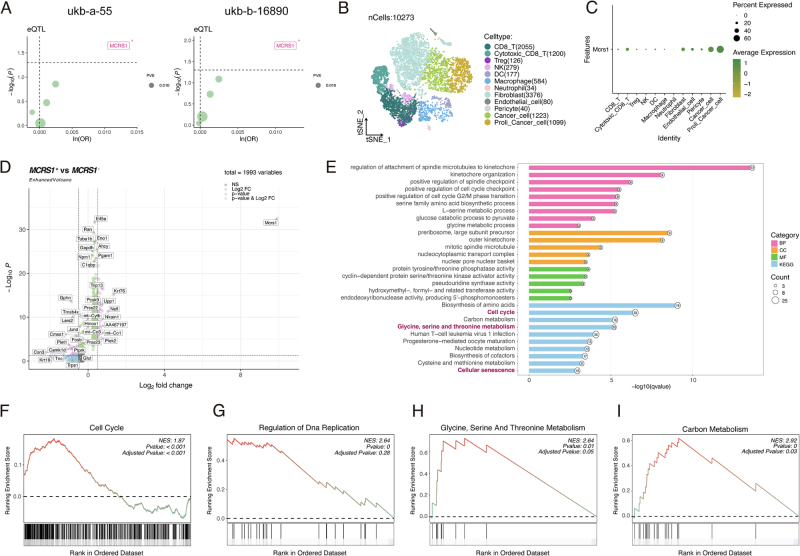
MCRS1 is a causal risk factor for breast cancer and drives a proliferative and metabolic program in cancer cells. (A) Two-sample Mendelian randomization (MR) analysis of the five hub genes. Volcano plots show that only genetically predicted expression of MCRS1 is significantly associated with an increased risk of breast cancer in two independent cohorts (ukb-a-55 and ukb-b-16 890). The *x*-axis represents the log odds ratio (ln(OR)), and the *y*-axis represents the −log10(*P*-value). (B) t-SNE plot of single-cell RNA sequencing data from an orthotopic E0771 murine breast tumor, showing distinct cell clusters within the tumor microenvironment. (C) Dot plot showing the expression of *Mcrs1* across the identified cell clusters. *Mcrs1* is predominantly expressed in “Cancer cells” and “Proliferating cancer cells.” Dot size indicates the percentage of cells expressing the gene; color indicates the average expression level. (D) Volcano plot showing differentially expressed genes between *Mcrs1*-positive and *Mcrs1*-negative cancer cells. (E) Functional enrichment analysis (GO and KEGG) of genes upregulated in *Mcrs1*-positive cancer cells. Enriched pathways are related to cell cycle, metabolism, and senescence. (F–I) GSEA enrichment plots for selected pathways in *Mcrs1*-positive versus *Mcrs1*-negative cancer cells, showing positive enrichment for (F) “Cell Cycle” (NES = 1.87, adjusted *P* < 0.001), (G) “Regulation of DNA Replication” (NES = 2.64, adjusted *P* = 0.28), (H) “Glycine, Serine and Threonine Metabolism” (NES = 2.64, adjusted *P* = 0.05), and (I) “Carbon Metabolism” (NES = 2.92, adjusted *P* = 0.03).

While the nomogram demonstrated strong correlative and diagnostic power, we sought to distinguish causal drivers from mere biomarkers among the five hub genes. To this end, we performed a two-sample MR analysis using large-scale genetic association data. The results of this causal inference analysis were striking: of the five candidates, only genetically predicted expression of MCRS1 was significantly associated with an increased risk of BC across two independent cohorts (Fig. 7A). The full results of the MR analyses for all five hub genes are provided in Supplemental Digital Content Table S1, available at: http://links.lww.com/JS9/G632. This pivotal finding identified MCRS1 as the primary causal risk factor among our hub genes.

To define the cellular context of MCRS1 function, we performed scRNA-seq on tumors from an orthotopic E0771 murine BC model. Unbiased clustering of the tumor microenvironment revealed that *Mcrs1* expression was highly enriched in the malignant cell populations, specifically “Cancer cells” and “Proliferating cancer cells” (Fig. 7B and C).

To elucidate the functional role of *Mcrs1* within cancer cells, we performed differential expression and pathway analyses by stratifying the cancer cell population into *Mcrs1*-positive and *Mcrs1*-negative subgroups (Fig. 7D). Genes upregulated in the *Mcrs1*-positive cells were significantly enriched in pathways related to cell cycle, metabolism (“Glycine, Serine and Threonine Metabolism”), and cellular senescence (Fig. 7E).

This finding was further substantiated by GSEA. A strong and statistically significant enrichment was observed for the “Cell Cycle” gene set (NES = 1.87, adjusted *P* < 0.001; Fig. 7F). Furthermore, we observed a strong positive enrichment trend for pathways related to DNA synthesis and metabolism, including “Regulation of DNA Replication” (NES = 2.64), “Glycine, Serine and Threonine Metabolism” (NES = 2.64), and “Carbon Metabolism” (NES = 2.92), although the adjusted *P*-values for these pathways were of borderline significance (Fig. 7G–I). Collectively, these results suggest that Mcrs1 expression in cancer cells is associated with a coordinated transcriptional program promoting proliferation and metabolic reprogramming.

### *MCRS1 is essential for BC progression both* in vitro *and* in vivo

**Figure 8.**
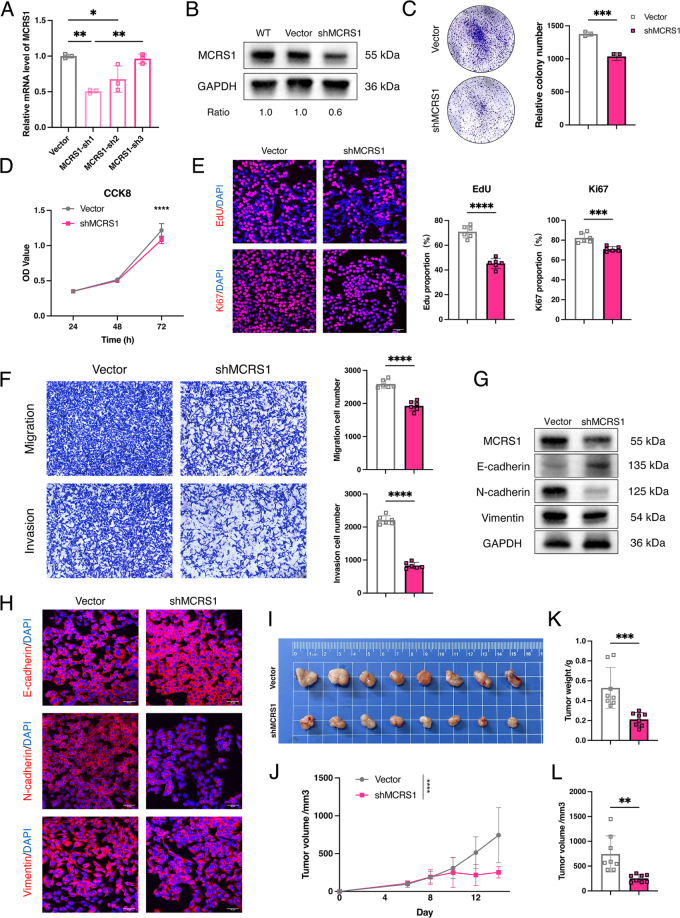
MCRS1 is required for breast cancer progression *in vitro* and *in vivo*. All experiments were performed using the E0771 murine breast cancer cell line stably transduced with a non-targeting vector or shRNA against *Mcrs1* (shMCRS1). (A, B) Validation of *Mcrs1* knockdown by (A) qPCR and (B) Western blot. (C) Colony formation assay showing reduced clonogenic survival in shMCRS1 cells. Representative images (left) and quantification (right). (D) CCK8 cell viability assay demonstrating impaired proliferation in shMCRS1 cells over 72 h. (E) EdU incorporation and Ki67 staining assays. Representative images (left) and quantification (right) showing a decreased proportion of proliferating shMCRS1 cells. (F) Transwell migration and invasion assays. Representative images (left) and quantification (right) showing reduced motility and invasiveness of shMCRS1 cells. (G, H) Analysis of EMT markers. (G) Western blot and (H) immunofluorescence staining showing increased E-cadherin and decreased N-cadherin and Vimentin in shMCRS1 cells. (I–L) Orthotopic xenograft tumor model in C57BL/6 mice (*n* =8 per group). (I) Representative images of excised tumors at the experimental endpoint. (J) Tumor growth curves over 12 days. (K) Final tumor weights. (L) Final tumor volumes. Knockdown of *Mcrs1* significantly suppressed tumor growth *in vivo*. Data in bar graphs are presented as mean ± SD. Statistical significance was determined by a two-tailed Student’s *t*-test. *P < 0.05, **P < 0.01, ***P < 0.001, ****P < 0.0001.

To experimentally validate the pro-tumorigenic role of MCRS1 predicted by our bioinformatic analyses, we performed a series of functional assays following stable knockdown of *Mcrs1* in the E0771 murine BC cell line. Knockdown efficiency was confirmed at both the mRNA and protein levels (Fig. 8A and B).

Consistent with our scRNA-seq analysis, depletion of *Mcrs1* resulted in a profound suppression of cell proliferation. *Mcrs1*-knockdown cells displayed a markedly impaired ability to form colonies and reduced viability in a CCK-8 assay (Fig. 8C and D). Furthermore, direct assessment of DNA synthesis and proliferation via EdU incorporation and Ki-67 staining, respectively, revealed a significant decrease in the percentage of positive cells following *Mcrs1* depletion (Fig. 8E).

Next, we investigated the function of MCRS1 in cell motility. Transwell assays demonstrated that *Mcrs1* knockdown significantly impaired both the migratory and invasive capacities of E0771 cells (Fig. 8F). To elucidate the underlying mechanism, we examined markers of the epithelial-to-mesenchymal transition (EMT). Western blot and immunofluorescence analyses revealed that *Mcrs1* depletion induced a mesenchymal-to-epithelial-like transition, characterized by a marked increase in the epithelial marker E-cadherin and a concomitant reduction in the mesenchymal markers N-cadherin and Vimentin (Fig. 8G and H).

Finally, to confirm the essential role of *Mcrs1* in tumor growth *in vivo*, we established an orthotopic model using the engineered cell lines. Consistent with our *in vitro* observations, tumors derived from *Mcrs1*-knockdown cells exhibited significantly attenuated growth compared to controls (Fig. 8I). This was evidenced by a slower tumor growth rate throughout the experiment and resulted in substantially smaller final tumor volumes and lower tumor weights at the experimental endpoint (Fig. 8J–L).

Taken together, these *in vitro* and *in vivo* experiments provide robust evidence that MCRS1 is a key functional driver of BC progression, promoting tumor cell proliferation, invasion, and overall tumor growth.

## Discussion

In this study, we sought to unravel the molecular nexus connecting three fundamental drivers of human disease: aging, MetS, and BC. Through a multi-layered analytical strategy that integrated transcriptomic data mining with causal inference and experimental validation, we identified and characterized MCRS1 as a core functional hub. Our findings collectively establish MCRS1 as a causal risk factor, a robust diagnostic biomarker, and a validated therapeutic target that mechanistically links age-associated metabolic dysregulation to BC progression.

Our investigation began with the unbiased observation that the BC transcriptome is defined by a striking dual signature: the concurrent upregulation of pathways related to cell cycle and senescence, and the downregulation of core metabolic processes. This finding, initially revealed by GSEA and subsequently validated by a network-based WGCNA approach in an independent cohort, provides strong support for the conceptual framework of the “Aging-Metabolism-Cancer Triangle.” While previous studies have extensively documented the role of accelerated cellular proliferation and metabolic reprogramming (i.e., the Warburg effect) in cancer^[58,59]^, our work highlights that these are not independent events but rather two facets of a single, coordinated program in BC. The simultaneous activation of senescence pathways, often considered a tumor-suppressive mechanism, alongside pro-proliferative pathways points to the complex and paradoxical role of senescence in cancer, where it can also promote tumorigenesis through the SASP^[16,60]^.

The central finding of our study is the identification of MCRS1 as the primary causal driver emerging from this complex molecular landscape. MCRS1 (Microspherule protein 1) is a protein lacking classical DNA-binding or enzymatic domains, primarily known as a scaffold component within larger epigenetic regulatory complexes such as INO80 and NSL, leaving its specific molecular function largely elusive^[61]^. The existing literature presents a confusing and even contradictory picture: while early studies linked MCRS1 to p53-dependent senescence^[62]^, its role in cancer has been ambiguous. Retrospective studies suggested its positive association with gastric cancer^[63]^, yet paradoxically, a functional study reported that MCRS1 inhibits gastric tumor cell migration and invasion^[64]^.

Our study significantly advances this understanding in several critical aspects. First, by employing MR, we elevate the role of MCRS1 in BC from a mere correlation to a causal risk factor, providing a much higher level of evidence. Second, our scRNA-seq analysis pinpoints the site of action, demonstrating that MCRS1 functions cell-autonomously within proliferating cancer cells, rather than as a general component of the tumor microenvironment. Most importantly, our scRNA-seq sub-cluster analysis creates a perfect mechanistic loop: MCRS1-positive cancer cells are transcriptionally programmed for the very same processes – heightened cell cycle, altered metabolism, and senescence – that we initially identified as the defining signature of bulk BC tissue. This provides a direct, cell-level link between the causal gene and the disease phenotype.

From a diagnostic perspective, the five-gene signature (MCRS1, MIF, PARP1, ADIPOR1, and ACD) operationalized into a nomogram demonstrated exceptional accuracy. Recognizing the risk of overfitting in single-cohort models, we validated our nomogram in an independent external dataset, where it maintained high discriminatory power with an AUC of 0.915. This external validation provides significant support for the robustness of our signature. While this represents a promising step toward a clinically relevant tool, we acknowledge that further validation in large, prospective, and diverse clinical cohorts with comprehensive metabolic profiling is still an essential prerequisite for any consideration of clinical implementation.

Despite the strengths of our integrative approach, this study has several limitations that warrant careful consideration. First, our discovery phase relied on publicly available datasets, some of which, particularly the MetS cohort, had limited sample sizes and lacked detailed clinical metadata. This may have constrained the initial scope of our signature, although the rigorous multi-step validation provides strong confidence in our lead candidate, MCRS1. Second, our causal inference for MCRS1 was limited to a single genetic instrument from whole-blood eQTLs – as no valid instruments were found in breast tissue data – precluding formal pleiotropy testing and requiring the assumption of a consistent effect across tissues. Third, there is a fundamental disconnect between our study’s conceptual framework and our experimental model. Our connection to aging was established computationally via cellular hallmark signatures, and our *in vivo* experiments were conducted in young, healthy mice. This design validates the fundamental oncogenic role of MCRS1 but does not directly test how its function is modulated by a systemically aged or metabolically dysregulated host. Finally, our functional conclusions are based on a single murine cell line. While the E0771 model is valuable, validating these findings across diverse human BC cell lines and patient-derived models is an essential next step to confirm the translational relevance and generalizability of our results. Similarly, the diagnostic performance of our five-gene nomogram, though successfully validated in one external cohort, requires further confirmation in large, prospective, and diverse clinical populations before any clinical application can be considered.

Furthermore, while our study robustly demonstrates what MCRS1 does – drive proliferation and invasion – the precise upstream and downstream mechanisms remain to be fully elucidated. Future investigations should aim to uncover how systemic signals of aging and metabolic stress (e.g., insulin and inflammatory cytokines) converge to regulate MCRS1 expression at the transcriptional or post-translational level. Given MCRS1’s established role as a component of epigenetic regulatory complexes like INO80 and NSL^[61]^, it is plausible that it executes its pro-tumorigenic function by modulating the chromatin landscape at the promoters of key cell cycle genes (e.g., Cyclins and CDKs) and master regulators of the EMT program (e.g., Snail and ZEB1). Identifying the direct downstream substrates and binding partners of MCRS1 in BC cells will be critical to fully understanding how it orchestrates this complex oncogenic program.

In conclusion, our study bridges a critical gap in our understanding of the interplay between aging, metabolism, and BC. We have identified MCRS1 as a central molecular node that causally links these three axes and represents a compelling candidate for future diagnostic and therapeutic investigation. This work provides a new lens through which to view BC pathogenesis and paves the way for mechanism-driven strategies to combat this deadly disease, especially in the context of an aging and increasingly metabolic-unhealthy population.

## Data Availability

The data that support the findings of this study have been deposited in CNSA with accession number CNP0008042. The scripts and command lines used in this study can be obtained from the lead contact upon request.
